# Calcipotriol counteracts betamethasone-induced decrease in extracellular matrix components related to skin atrophy

**DOI:** 10.1007/s00403-014-1485-3

**Published:** 2014-07-16

**Authors:** Hanne Norsgaard, Sandrine Kurdykowski, Pascal Descargues, Tatiana Gonzalez, Troels Marstrand, Georg Dünstl, Mads Røpke

**Affiliations:** 1 Department of Molecular Biomedicine, LEO Pharma A/S, Industriparken 55, Ballerup, Denmark; 2 Disease Pharmacology, LEO Pharma A/S, Industriparken 55, Ballerup, Denmark; 3 External Discovery, LEO Pharma A/S, Industriparken 55, Ballerup, Denmark; 4 Clinical Pharmacology, LEO Pharma A/S, Industriparken 55, Ballerup, Denmark; 5 Genoskin, Oncopole, 1 Place Pierre Potier, Toulouse, France

**Keywords:** Betamethasone, Calcipotriol, Epidermal thinning, Extracellular matrix, Skin atrophy

## Abstract

The calcipotriol/betamethasone dipropionate fixed-combination gel is widely used for topical treatment of psoriasis vulgaris. It has been hypothesized that calcipotriol counteracts glucocorticoid-induced skin atrophy which is associated with changes in the extracellular matrix (ECM). To elucidate the combined effects of calcipotriol and betamethasone on key ECM components, a comparative study to the respective mono-treatments was carried out. The effect on collagen I synthesis, matrix metalloproteinase (MMP) secretion, and hyaluronic acid (HA) production was investigated in primary human fibroblast and keratinocyte cultures as well as in a human skin explant model. We show that calcipotriol counteracts betamethasone-induced suppression of collagen I synthesis. Similarly, calcipotriol and betamethasone have opposing effects on MMP expression in both fibroblasts and keratinocytes. Moreover, calcipotriol is able to restore betamethasone-impaired HA synthesis in keratinocytes and prevent betamethasone-induced epidermal thinning in minipigs upon treatment with the calcipotriol/betamethasone gel. In summary, our results show for the first time in primary human skin cultures that calcipotriol reduces early signs of betamethasone-induced skin atrophy by modulation of key ECM components. These results indicate that the calcipotriol component of the fixed-combination gel counteracts the atrophogenic effects of betamethasone on the skin.

**Electronic supplementary material:**

The online version of this article (doi:10.1007/s00403-014-1485-3) contains supplementary material, which is available to authorized users.

## Introduction

Topical use of glucocorticoids can lead to the development of skin atrophy which is characterized by reduced skin thickness and elasticity, telangiectasia and purpura [3]. Glucocorticoids have been shown to reduce the proliferation and size of keratinocytes and to impair the skin barrier through inhibition of lipid synthesis [7, 15, 17]. Glucocorticoids also decrease the proliferation of fibroblasts and the production of extracellular matrix (ECM) proteins [13, 27]. Importantly, glucocorticoids inhibit collagen synthesis in fibroblasts and reduce the expression and activity of collagenases and other matrix metalloproteinases (MMPs) in keratinocytes and fibroblasts [2, 4, 18, 23, 28]. Furthermore, through regulation of hyaluronan synthase (HAS)-2 in fibroblasts and keratinocytes, glucocorticoids decrease the level of hyaluronic acid (HA), a glycosaminoglycan which plays an important role in maintaining dermal and epidermal structure, flexibility and water binding capacity [1, 8, 26, 34]. By affecting important ECM components, glucocorticoids restrict the dynamic remodeling of the skin, thus contributing to skin atrophy.

In contrast, studies mostly performed in mice suggest that vitamin D receptor (VDR) agonists have opposing effects on many of the cellular and molecular mechanisms underlying glucocorticoid-induced skin atrophy, including effects on cell proliferation, epidermal lipids and antimicrobial peptides [9–11, 14]. Combination of betamethasone with calcipotriol has proven to be an effective topical treatment of psoriasis vulgaris that can be used for up to a year with very low incidence of skin atrophy [19]. These observations suggest that combination with a VDR agonist has the potential to mitigate the unwanted cutaneous side effects associated with topical glucocorticoid mono-therapy [29]. The present study is the first to investigate the combined effects of vitamin D agonists and glucocorticoids on key ECM components at the cellular and molecular level in primary human skin cultures.

## Materials and methods

### Test reagents

Mono-components of the fixed-combination gel (Daivobet/Dovobet^®^) containing betamethasone dipropionate (0.5 mg/g) and calcipotriol (50 µg/g) were produced in the corresponding gel vehicle at LEO Pharma. Stock solutions of calcipotriol (MC 00903; LEO Pharma) and betamethasone dipropionate (provided by LEO Pharma) with a concentration of 10 mM were prepared in DMSO. The stock solutions were diluted in cell culture medium at the time of use.

### Cell cultures

Cultures of primary human dermal fibroblasts (Invitrogen Life Technologies, Carlsbad, CA, USA) were propagated in M106 medium supplemented with Low Serum Growth Supplement consisting of 1.9 % fetal bovine serum (FBS), 1 µg/mL hydrocortisone, 10 ng/mL human epidermal growth factor (EGF), 3 ng/mL basic fibroblast growth factor (bFGF), 10 µg/mL heparin. Furthermore, gentamycin was added to the culture medium. Cells were incubated in a humidified atmosphere of 5 % CO_2_ at 37 °C. The medium was changed the day before and on the day of treatment (90 % confluent cultures) to M106 medium containing 1.9 % charcoal-treated FBS and hydrocortisone was omitted from the supplement to appropriately assess the effect of betamethasone dipropionate. Supplementation with the other factors mentioned above is needed to maintain proliferative cultures with normal fibroblast morphology.

Cultures of primary human epidermal keratinocytes (Invitrogen Life Technologies, Carlsbad, CA, USA) were grown in EpiLife medium supplemented with 0.2 ng/mL human EGF, 0.2 % bovine pituitary extract (BPE), 5 µg/mL bovine insulin, 5 µg/mL bovine transferrin, 0.18 µg/mL hydrocortisone and gentamycin. Cells were incubated in a humidified atmosphere of 5 % CO_2_ at 37 °C. The medium was changed the day before and on the day of treatment (80 % confluent cultures) to EpiLife medium without any supplement and containing 1.2 mM calcium to induce differentiation. Keratinocytes can be maintained in non-supplemented medium for a few days without loss of viability.

Cell cultures were treated with the compounds in duplicate for the indicated time points. The final concentration of the compounds was 100 nM for calcipotriol and 1 µM for betamethasone both for single treatments and for the combination. These concentrations are based on exposure in ex vivo pig and human skin after topical application of the calcipotriol/betamethasone fixed-combination product. Supernatants from fibroblast and keratinocyte cultures were collected for analysis of secreted proteins. Four independent experiments were performed using fibroblasts from two different donors. In three of the experiments, fibroblast cultures were processed for RNA purification and qPCR analysis. Five independent experiments using keratinocytes from three different donors were performed.

### Quantitative real-time PCR

Total RNA was extracted from cells using the RNeasy mini kit (Qiagen, Germantown, MD, USA) according to the instructions provided. cDNA synthesis was performed with the High-Capacity cDNA Reverse Transcription Kit (Applied Biosystems, Foster City, CA, USA). 2.5 µL of cDNA (equivalent to 5 ng RNA) from each sample was amplified in a total volume of 10 µL by quantitative real-time PCR using Taqman^®^ Gene Expression Assays (*COL1A1*-Hs00164004_m1, *HAS2*-Hs00193435_m1, *GAPDH*-Hs99999905_m1) and PRISM7900HT sequence detection system (SDS 2.3) from Applied Biosystems. *GAPDH* was used for normalization as it was found to be a stable reference gene not affected by calcipotriol or betamethasone treatment in human dermal fibroblasts.

### Immunoassays

Analysis of secreted CICP (C-terminal pro-peptide of type I collagen) in cell culture supernatants from fibroblasts was performed using a human CICP EIA kit (Quidel, San Diego, CA, USA). Detection of MMP-1 and MMP-3 in cell culture supernatants from fibroblasts and keratinocytes was carried out by the use of a human MMP 3-plex ultra-sensitive kit and measured on a MSD platform (Meso Scale Discovery, Gaithersburg, MD, USA). Hyaluronic acid (HA) was measured in the cell culture supernatants from fibroblasts and keratinocytes by a human HA competitive ELISA kit (Echelon Biosciences, Salt Lake City, UT, USA).

### Production and culture of NativeSkin^®^ models

Genoskin collected anonymized human skin samples from donors that underwent abdominoplasty procedure and had given their written informed consent. Donors did not have any dermatological disorders and did not use glucocorticoid treatment. Full ethical approval for the study protocol was obtained from the French ethical research committee (Comité de Protection des Personnes) and authorization was given from the French ministry of Research. All studies were conducted according to the Declaration of Helsinki protocols.

Immediately following surgery, skin samples were transported at 4 °C before being processed to produce NativeSkin^®^ models. Subcutaneous adipose tissue was removed from the skin sample. 8 mm punch biopsies were excised and embedded in a proprietary fibrin-based matrix in transwells (Filter pore size 1 µm, Millicell). The epidermal surface of skin biopsies was left in contact with the air and the dermal compartment was immersed in the matrix. NativeSkin^®^ models were cultured in 12-well plates in a proprietary and chemically-defined hydrocortisone- and serum-free medium supplemented with 100 μg/mL penicillin and 100 μg/mL streptomycin in a humidified atmosphere of 5 % CO_2_ at 37 °C. The medium was changed every day. 10 µL of formulation was applied on the epidermal surface using a positive displacement pipette once daily for 6 days. Before each re-application of formulation, any remaining formulation was removed with a cotton swab.

### Histological and immunofluorescence analyses

Treated NativeSkin^®^ models were fixed in 10 % neutral-buffered formalin and embedded in paraffin wax. 5 µm cross-sections were stained with hematoxylin–eosin or anti-pro-COLA1 (MAB1912, Merck Millipore, Billerica, MA, USA), anti-MMP-1 (EP1237Y, ab52631, Abcam, Cambridge, UK), anti-MMP-3 (ab53015, Abcam) and anti-HAS-2 (ab140671, Abcam) antibodies. Skin sections were stored at 60 °C for 1 h prior to incubation with antibodies for 1 h. A specific signal was detected using secondary antibodies conjugated to Alexa Fluor 555 dye (Invitrogen Life Technologies, Carlsbad, CA, USA). DAPI (D9542, Sigma-Aldrich, St. Louis, MO, USA) was used to counterstain skin sections for the immunofluorescence analyses. Images were obtained with a Nikon Eclipse 80i fluorescence microscope and its dedicated NIS-Element AR software. We made sure that pixel intensity was not saturated by turning on the pixel saturation indication button, provided in the NIS-Element AR software. All images for a respective ECM marker were obtained with strictly the same parameters such as signal intensity and duration of exposure. Signals were quantified by analyzing images with the ImageJ software and expressed in arbitrary units of fluorescence corresponding to the sum of the gray values of all the pixels in the selection divided by the number of pixels. Results are normalized relative to vehicle controls.

### Minipig study

Eight five-month-old female Göttingen minipigs (9–11 kg bodyweight) were purchased from Ellegaard Göttingen minipigs, Denmark. Animals were group-housed with unlimited access to food and water and acclimatized for 2 weeks before start of study. Animals were anaesthetized with a mixture of zoletil, xylazine, ketamine and butorphanol i.v. Ten test fields of 6.25 cm^2^ each (on the back and behind the ears) were delineated with tattoo. A 5-mm punch biopsy was taken from the upper left corner of each test field and the wound was closed with a metal clip. 80 µL of the designated formulation was applied topically to the test field Monday to Friday for a total period of 4 weeks. The animals were observed daily and any sign of local skin reaction was noted. Treatment of the test fields occurred in a randomized manner. At the end of the study all animals were euthanized by an overdose of pentobarbital i.v. and full-thickness test areas were excised. All animal procedures were conducted in accordance with institutional guidelines and licensed by the Danish Animal Experiments Inspectorate. A 5-mm punch biopsy was obtained from each area and fixed in 4 % formaldehyde, embedded in paraffin, sectioned at 5 µm and stained with Masson’s trichrome. Slides were analyzed using Visiomorph™ system version 4.3.2.0 (Visiopharm, Hørsholm, Denmark). The mean epidermal thickness (MET) was determined by dividing the total epidermal area with the epidermal surface length.

### Statistical analysis

For in vitro studies, statistical comparison between groups was performed by one-way ANOVA followed by Tukey’s multiple comparison test using GraphPad Prism v. 5.0 (GraphPad, San Diego, CA, USA). For the minipig study, a mixed effects model was fitted to the epidermal thickness measurements, with thickness as a fixed effect and animal and treatment field as random effects. The vehicle group was used as reference for testing significance of treatment using R version 2.15.2.

## Results

### Calcipotriol counteracts betamethasone-induced suppression of ECM components in primary human fibroblasts and keratinocytes in vitro

**Fig. 1:**
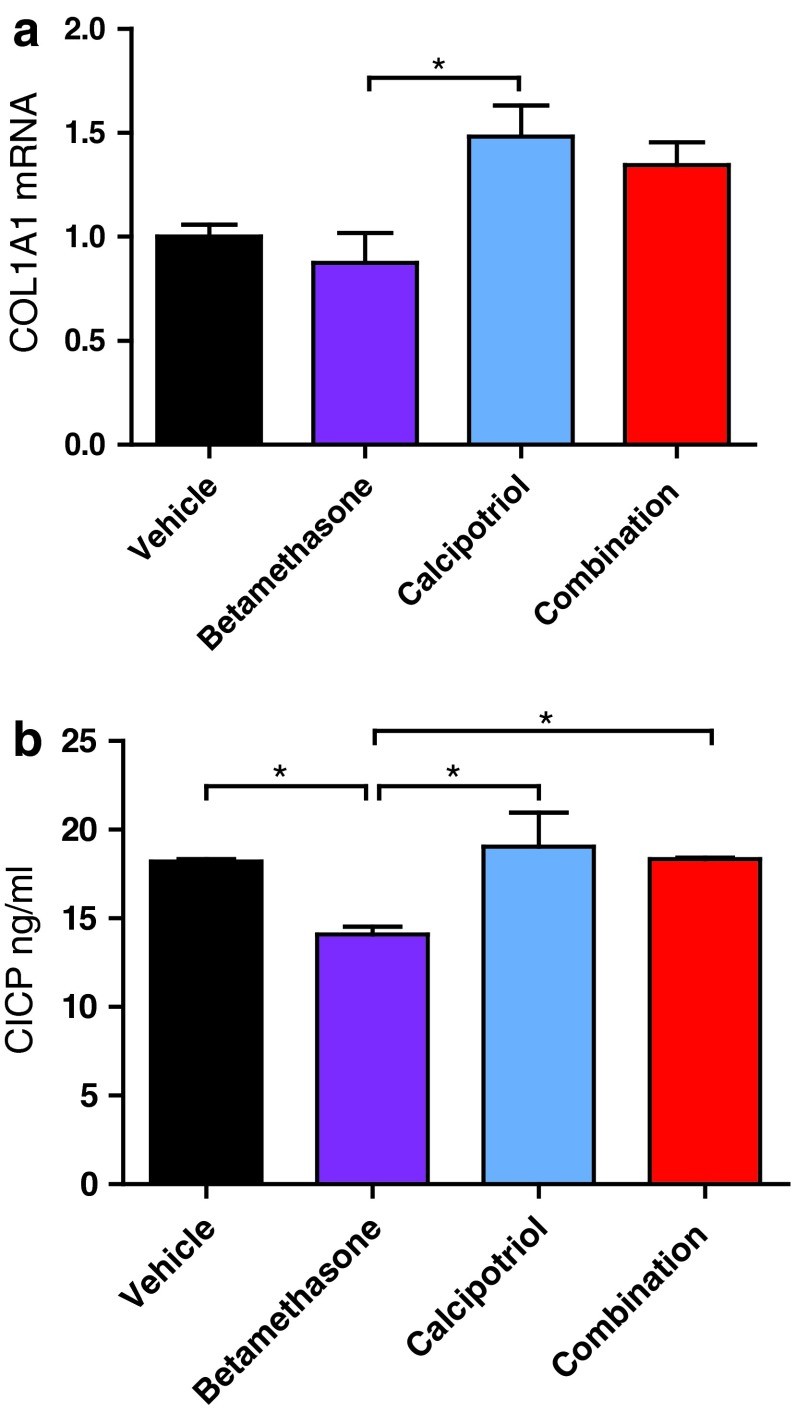
Collagen I synthesis is not decreased by combination treatment with betamethasone and calcipotriol. Cultures of human dermal fibroblasts were treated in duplicates with 100 nM calcipotriol, 1 µM betamethasone, 100 nM calcipotriol + 1 µM betamethasone or a corresponding vehicle control (0.1 % DMSO). **a** *COL1A1* mRNA expression was analyzed by qPCR after 24 h of treatment and normalized to *GAPDH* and **b** CICP (C-terminal pro-peptide of type I collagen) was measured by ELISA in culture supernatants after 48 h of treatment. The values ± SD are representative of three (**a**) and four (**b**) independent experiments using two donors. *P < 0.05 (one-way ANOVA test)

**Fig. 2:**
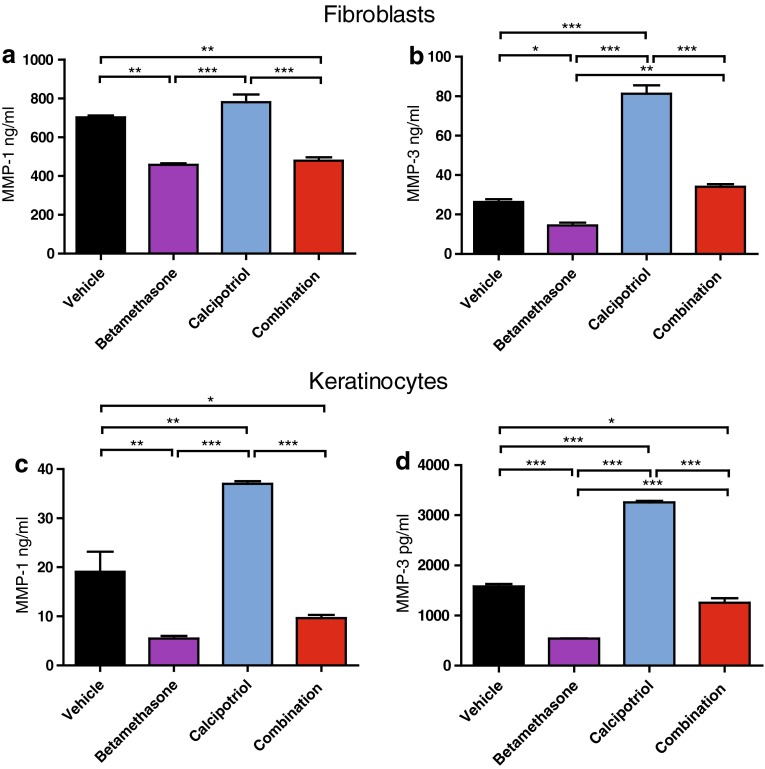
Calcipotriol and betamethasone have opposing effects on MMP expression. Cultures of human dermal fibroblasts and human epidermal keratinocytes (in the presence of 1.2 mM calcium) were treated as described in Fig. 1 and analyzed for levels of MMP-1 and MMP-3 in culture supernatants after 48 h of treatment using a MSD kit. **a** MMP-1 and **b** MMP-3 levels detected from fibroblast cultures and **c** MMP-1 and **d** MMP-3 levels detected from keratinocyte cultures. The values ± SD are representative of four independent experiments using two donors (**a**, **b**) and five independent experiments using three donors (**c**, **d**). *P < 0.05; **P < 0.01; ***P < 0.001 (one-way ANOVA test)

**Fig. 3:**
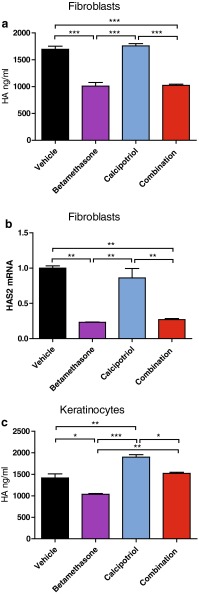
Betamethasone-impaired production of HA is counteracted by calcipotriol in keratinocytes but not in fibroblasts. Cultures of human dermal fibroblasts and human epidermal keratinocytes (in the presence of 1.2 mM calcium) were treated as described in Fig. 1. HA was measured by ELISA in supernatants from **a** fibroblast and **c** keratinocyte cultures after 48 h of treatment and **b** *HAS2* mRNA expression was analyzed by qPCR after 24 h of treatment and normalized to *GAPDH*. The values ± SD are representative of four (**a**) and three (**b**) independent experiments using two donors and **c** five independent experiments using three donors. *P < 0.05; **P < 0.01; ***P < 0.001 (one-way ANOVA test)

To investigate the effects of betamethasone and calcipotriol on ECM components, cultures of primary human dermal fibroblasts, and differentiated primary human epidermal keratinocytes were treated with calcipotriol (100 nM), betamethasone (1 µM), calcipotriol in combination with betamethasone (100 nM/1 µM; ratio 1:10 as in the fixed-combination product) or a corresponding vehicle control (0.1 % DMSO). Final concentrations of calcipotriol and betamethasone used in this study are based on exposure in ex vivo pig and human skin after topical application of the calcipotriol/betamethasone fixed-combination product. No compound-induced changes in cell morphology or confluence were observed in comparison with the vehicle control. The extracellular protein collagen I, matrix metalloproteinases MMP-1 and MMP-3, and the glycosaminoglycan hyaluronic acid were chosen as representative ECM components to be analyzed in this study. Gene expression and protein analysis using cells from different donors were performed to address the treatment effect on ECM components. Overall comparable treatment effects were found at 24 and 48 h and across different donors as presented in Supplementary Figs. 1 and 2. Representative experiments are shown in Figs. 1, 2 and 3.

For collagen I, which is synthesized by fibroblasts as pro-collagen and processed to mature collagen (tropo-collagen) by cleavage of N- and C-terminal pro-peptides, *COL1A1* mRNA and secretion of the C-terminal pro-peptide CICP were chosen as readouts. In the fibroblast cultures, *COL1A1* mRNA expression was slightly decreased by betamethasone but moderately increased by calcipotriol with a statistically significant difference between the two mono-treatments (Fig. 1a). *COL1A1* mRNA expression was comparable in fibroblasts treated with either calcipotriol alone or the calcipotriol/betamethasone combination. Furthermore, the compiled data from several independent experiments showed a significant difference between betamethasone and the combination treatment (Supplementary Fig. 1a). Calcipotriol and betamethasone also showed opposing effects on CICP as secreted levels were significantly reduced by treatment with betamethasone but remained unaffected by calcipotriol (Fig. 1b). Again, combination of calcipotriol with betamethasone resulted in levels of CICP that were comparable to the vehicle control and significantly higher than those observed for betamethasone mono-treatment.

Matrix metalloproteinases are ECM-degrading enzymes produced by fibroblasts and keratinocytes that are of critical importance for ECM homeostasis in the skin. Treatment with betamethasone alone resulted in a reduced secretion of both MMP-1 and -3 in fibroblast as well as keratinocyte cultures (Fig. 2a–d). In contrast, secretion of MMP-3 was markedly increased by calcipotriol in both fibroblasts and keratinocytes (Fig. 2b, d). Similarly, treatment with calcipotriol significantly increased MMP-1 secretion in keratinocytes (Fig. 2c); however, little to no increase of MMP-1 was observed with calcipotriol in fibroblasts (Fig. 2a; Supplementary Fig. 1c). Again, when combined with betamethasone, calcipotriol managed to counteract glucocorticoid-induced suppression of MMP-3 secretion in fibroblasts as well as keratinocytes (Fig. 2b, d). The counteracting effect of calcipotriol in combination with betamethasone is further supported by the findings for MMP-1, showing a partial effect in keratinocytes (Fig. 2c) which was significantly different from betamethasone mono-treatment based on analysis of compiled data from independent experiments (Supplementary Fig. 2a). In contrast, combination of calcipotriol with betamethasone did not increase MMP-1 secretion in fibroblasts in line with the minimal effect of calcipotriol mono-treatment (Fig. 2a).

The glycosaminoglycan HA is synthesized by three different hyaluronan synthases (HAS)-1, -2, and -3 expressed in fibroblasts as well as keratinocytes. In the present study, HA synthesis was monitored by determination of secreted HA in cell culture supernatants. Additionally, cellular expression of *HAS2* mRNA was analyzed. In this setting, treatment with betamethasone alone significantly reduced HA secretion from fibroblasts as well as keratinocytes (Fig. 3a, c). In contrast, HA secretion from fibroblasts remained unaffected by calcipotriol mono-treatment. In the combination, calcipotriol was not able to counteract the betamethasone-induced suppression of HA secretion in fibroblasts in line with the lack of effect by calcipotriol mono-treatment (Fig. 3a). Analysis of *HAS2* mRNA expression in fibroblasts showed a picture similar to that observed for HA secretion, i.e. comparable levels for vehicle and calcipotriol treatment and a decrease in *HAS2* mRNA expression by betamethasone and the calcipotriol/betamethasone combination (Fig. 3b). In keratinocytes, however, calcipotriol alone significantly increased HA secretion and was able to fully counteract the glucocorticoid-induced inhibition of HA secretion when combined with betamethasone (Fig. 3c).

### The counteracting effect of calcipotriol on betamethasone-induced decrease in ECM proteins can be translated to a human skin explant model

**Fig. 4:**
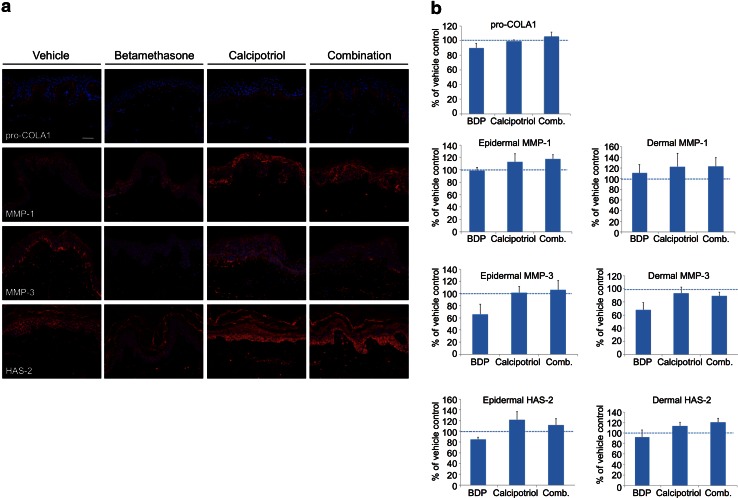
Calcipotriol counteracts betamethasone-induced decrease in ECM proteins upon topical application of the fixed-combination gel in a human skin explant model. **a** Immunostaining of skin cross-sections with anti-pro-COL1A1, anti-MMP-1, anti-MMP-3 and anti-HAS-2 antibodies after 6 days of daily topical application of the vehicle gel, the fixed-combination gel, and the mono-components in the corresponding vehicle gel. Three replicates were used for each treatment. Signals were visualized with a fluorescence microscope. *Scale bar* is 50 µm. **b** Quantifications of immunostainings performed with ImageJ software. Values in the *histograms* are normalized relative to vehicle controls

We next investigated if the findings from in vitro cultures could be translated to ex vivo human skin subjected to repeated topical application of the calcipotriol/betamethasone gel. For this purpose, we used a recently developed skin explant model (NativeSkin^®^) which consists of full-thickness human skin biopsies that are embedded in a proprietary fibrin-based matrix in transwells and cultured in hydrocortisone- and serum-free medium (Supplementary Fig. 3). Histological features of normal skin are maintained during ex vivo culture of the NativeSkin^®^ model for up to 1 week (Supplementary Fig. 4). Furthermore, repeated daily topical application of the vehicle gel for 6 days did not affect skin morphology and epidermal layers demonstrating that the vehicle gel was well tolerated in this skin model (Supplementary Fig. 5). Therefore, this treatment regimen was used in the NativeSkin^®^ model to investigate the expression of pro-collagen I, MMP-1, MMP-3, and HAS-2 by quantitative immunofluorescence analysis following topical application of gel formulations containing betamethasone dipropionate, calcipotriol and the fixed-combination of calcipotriol and betamethasone dipropionate, using the corresponding vehicle gel as a control. A representative example of two independent experiments in different donors is shown in Fig. 4.

Pro-collagen I immunoreactivity was found in the upper papillary dermis in conjunction with the stratum basale in vehicle-treated samples (Fig. 4a). Betamethasone mono-treatment resulted in a mild reduction of pro-collagen I expression compared with the vehicle gel (Fig. 4a, b). Treatment with calcipotriol alone or with the calcipotriol/betamethasone gel resulted in levels of pro-collagen I comparable to the vehicle control indicating that calcipotriol was able to prevent the betamethasone-induced suppression of pro-collagen I. Thus, the counteracting effect of calcipotriol on betamethasone-induced suppression of collagen I synthesis found in vitro could be translated to human skin explants treated with the calcipotriol/betamethasone gel.

Immunoreactivity of MMP-1 and MMP-3 was present in all viable layers of the epidermis and detected in some dermal fibroblasts in vehicle-treated samples (Fig. 4a). Betamethasone mono-treatment did not affect expression of MMP-1 whereas expression of MMP-3 was clearly suppressed (Fig. 4a, b). Calcipotriol mono-treatment slightly enhanced the expression of MMP-1 and resulted in comparable levels of MMP-3 as found in the vehicle control. Treatment with the calcipotriol/betamethasone gel showed that calcipotriol could still increase epidermal expression of MMP-1 even in the presence of betamethasone and counteracted the betamethasone-induced decrease of MMP-3. In line with in vitro results from monolayer cultures, betamethasone and calcipotriol showed opposing effects on MMP expression in the human skin explant model.

HAS-2 immunostaining was detected in all layers of the epidermis and in some dermal fibroblasts in vehicle-treated samples (Fig. 4a). The epidermal staining of HAS-2 was reduced by betamethasone mono-treatment whereas no difference was detected in the dermis (Fig. 4a, b). Calcipotriol mono-treatment resulted in slightly elevated levels of epidermal HAS-2 as compared with the vehicle control. Treatment with the calcipotriol/betamethasone gel showed that calcipotriol could restore betamethasone-decreased HAS-2 expression to levels comparable to the vehicle control. This is in line with the in vitro data on HA secretion where calcipotriol was able to counteract betamethasone-induced reduction of HA in keratinocytes. At the investigated time point, none of the treatments induced epidermal or dermal thinning based on Masson Trichrome-stained sections (data not shown). This result is consistent with clinical findings demonstrating skin thinning as a long-term treatment effect.

### Calcipotriol prevents betamethasone-induced epidermal thinning in minipigs correlating to HA modulation

**Fig. 5:**
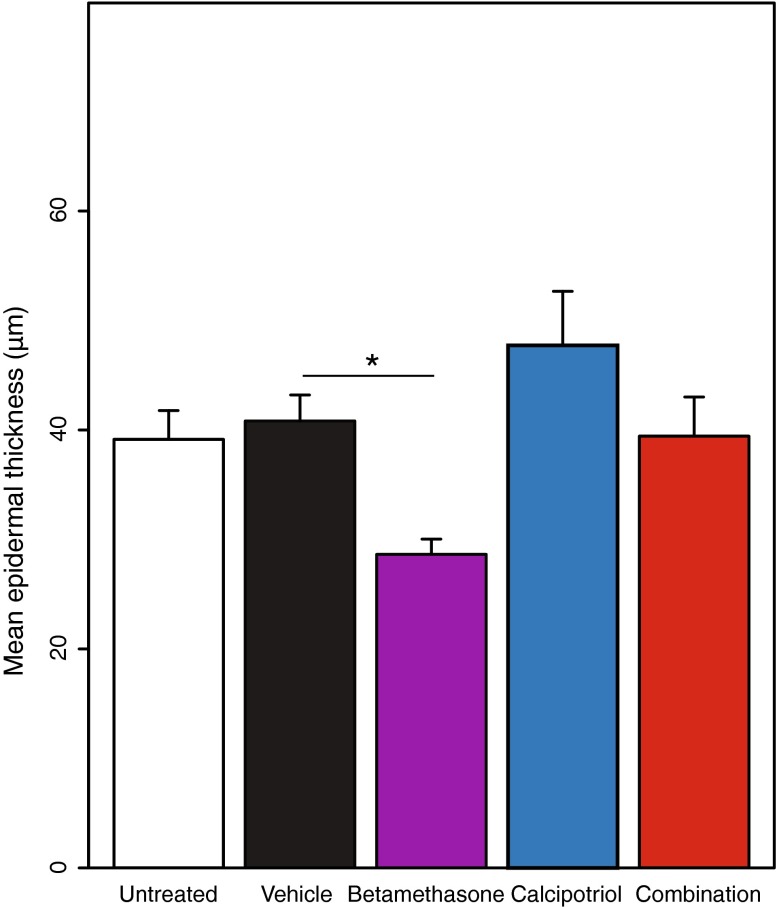
Calcipotriol prevents betamethasone-induced epidermal thinning in minipigs. Göttingen minipigs were dosed topically with the vehicle gel, the calcipotriol/betamethasone gel, or the mono-components in the corresponding vehicle gel for a total of 4 weeks. Epidermal thickness was analyzed on Masson’s trichrome-stained sections (mean ± SD). The vehicle group was used as reference for testing significance of treatment by a mixed effects model with thickness as a fixed effect and animal and treatment field as random effects (R version 2.15.2). *P < 0.05

Levels of HA in the skin may affect keratinocyte proliferation and epidermal thickness. Therefore, we investigated whether the differential effects of betamethasone and calcipotriol on HA observed in keratinocytes would translate into effects on epidermal thickness in vivo. For this purpose, minipigs were used to investigate drug-induced effects on epidermal thickness following topical treatment for a total of 4 weeks with vehicle gel, the calcipotriol/betamethasone gel, or the mono-components in the corresponding vehicle gel. Upon termination, mean epidermal thickness was analyzed on Masson’s trichrome-stained sections and found to be similar in the untreated and vehicle-treated group (Fig. 5). In contrast, mean epidermal thickness was significantly reduced by treatment with betamethasone but modestly increased by calcipotriol (however, the effect by calcipotriol was not significantly different from the vehicle group). Thus, the decreased HA synthesis in keratinocytes induced by betamethasone in vitro and ex vivo seems to correlate with reduced epidermal thickness in minipigs in vivo. Similarly, the increased HA synthesis induced by calcipotriol in vitro and ex vivo seems to correlate with a modest increase in epidermal thickness in vivo. Finally, in accordance with the in vitro and ex vivo data on HA modulation, treatment with the calcipotriol/betamethasone gel maintained epidermal thickness comparable to the controls in minipigs, showing that calcipotriol was able to prevent betamethasone-induced epidermal thinning.

## Discussion

The effects of glucocorticoids on extracellular matrix components have been studied in monolayer cultures of human dermal fibroblasts by several groups, however, with rather divergent results [1, 28, 33]. In one study, treatment with glucocorticoids for one to five days showed no effect on *COL1A1* and *COL3A1* mRNA expression while *HAS2* mRNA expression and HA production were significantly suppressed [1]. In another study, treatment with glucocorticoids for 24 h did not affect *COL1A1* and *COL3A1* mRNA expression in human or rat dermal fibroblasts. In contrast, the mRNA expression for these genes was strongly suppressed in 3T3 mouse embryonic fibroblasts [28], suggesting dependent differences. However, Weindl et al. [33] demonstrated a marked downregulation of *COL1A1*, less inhibition of *COL3A1* but no effects on *COL1A2*, *COL4A1* and *MMP1* mRNA expression in human dermal fibroblasts. Possible explanations for these apparently contradicting findings may relate to the assay conditions and more specifically the addition of serum to the assay media. In the two first studies [1, 28], glucocorticoids were tested in human dermal fibroblast cultures supplemented with serum (at which concentration and whether charcoal-treated serum was used is not specified in the publications). The third study was apparently conducted without supplementation of the culture medium with serum or growth factors during the experiment [33]. The lack of serum may significantly impair the physiological responses of fibroblasts with noticeable changes in cell proliferation and morphology. Such variations in culture conditions may well explain the differing results. Using our growth conditions (1.9 % charcoal-treated serum plus supplementation as described), we find that treatment with glucocorticoids result in detectable but modest effects on *COL1A1* mRNA expression and CICP secretion whereas stronger suppression is seen on *HAS2* mRNA expression and secretion of HA, MMP-1 and MMP-3.

As monolayer cultures lack organotypic features, mechanisms of glucocorticoid-induced skin atrophy have been explored in reconstructed human full-thickness skin models. In studies using the Phenion-FT skin model (Henkel KGaA), a marked decrease in collagen I synthesis and reduction in epidermal layers was observed by addition of glucocorticoids to the culture medium as well as by topical application for 11–14 days [28, 35]. However, the fact that Zoller et al. [35] reported up to 20–30 % reduction in epidermal layers of untreated or vehicle controls during this extended treatment period suggests a possible confounding effect. In contrast to these findings, another study using the EpiDerm FT skin model (MatTek Corp.) reported no effect of glucocorticoids on mRNA expression of *COL1A1* and *MMP1* and on epidermal thickness after topical application for 7 days [33]. The seemingly contradictive results from these studies may be caused by the different treatment schedules but also by the specific features of these skin models. In our experience, the Phenion-FT model has higher mechanical stability and contains more epidermal layers than the EpiDerm FT skin model.

Some drawbacks of reconstructed skin models relate to their highly permeable skin barrier compared to normal skin and the morphological changes and decrease in epidermal layers typically seen during prolonged culture and upon repeated topical application of vehicles. Due to these confounders, the use of human skin explants may offer a more relevant option in terms of translation to human skin in vivo. Here, we use the NativeSkin^®^ model containing full-thickness human skin embedded in a proprietary matrix. Compared to human skin explants using standard culture conditions, the NativeSkin^®^ model provides the opportunity to perform repeated daily topical application up to a week without affecting skin morphology and epidermal layers of the explant (Supplementary Fig. 5).

In the present study we have used several different readouts to assess the impact of betamethasone on collagen I synthesis. We show that betamethasone treatment for 24 and 48 h resulted in a minor inhibition of *COL1A1* mRNA but a significant decrease of CICP secretion in primary human dermal fibroblasts in vitro. Furthermore, immunofluorescence analysis shows that betamethasone treatment reduced pro-collagen I in the NativeSkin^®^ explant model. In addition to the effect on collagen I synthesis, betamethasone treatment significantly reduced the secretion of MMP-1 in both fibroblasts and keratinocytes in vitro. These findings are in line with published studies and support the concept that glucocorticoids restrict remodeling and adaptation of the skin by diminishing collagen synthesis and collagenase (MMP-1) production [2, 4, 5, 20, 24, 28]. Likewise, MMP-3 secretion was significantly decreased by betamethasone treatment in both fibroblasts and keratinocytes in vitro as well as in the human skin explant model. In contrast, MMP-1 expression in the human skin explant model was unaffected by betamethasone treatment at the investigated time point.

Previous studies in hairless mice have suggested that vitamin D receptor agonists may counteract glucocorticoid-induced skin atrophy and induce collagen synthesis [10, 11]. However, a potential confounder of these studies is the use of the highly potent vitamin D analog (KH1060) which is known to induce skin irritation and skin thickening in mice [11]. In the present study we show that calcipotriol was able to prevent betamethasone-induced suppression of collagen I synthesis in human dermal fibroblasts in vitro as well as in the human skin explant model treated with the calcipotriol/betamethasone gel. The differential effects of betamethasone and calcipotriol on collagen I synthesis could maybe be explained by differential effects on TGF-beta which positively regulates the expression of the *COL1A1* gene [32]. Glucocorticoids have been found to decrease TGF-beta expression whereas vitamin D has been shown to increase TGF-beta expression in both dermal fibroblasts and keratinocytes [12, 21, 25].

Vitamin D has previously been shown to increase the expression of MMP-1 and MMP-3 in human synovial fibroblasts [30]. To our knowledge, there are no published studies addressing the effect of vitamin D on MMP expression in keratinocytes. Our in vitro results demonstrate that calcipotriol enhanced levels of MMP-3 in dermal fibroblasts and in keratinocytes whereas calcipotriol only significantly augmented MMP-1 levels in keratinocytes but not in dermal fibroblasts. In the human skin explant model, calcipotriol maintained levels of MMP-1 and MMP-3 even in the presence of betamethasone upon treatment with the calcipotriol/betamethasone gel.

Glucocorticoids have been shown to decrease *HAS2* mRNA expression as well as production of HA in fibroblasts and keratinocytes in vitro and to reduce dermal HA levels in human skin in vivo [8]. Our results confirm that betamethasone treatment impairs both *HAS2* expression and HA secretion in vitro. Furthermore, we show that the decreased HA synthesis induced by betamethasone in keratinocytes in vitro and in the human skin explant model translates to reduced epidermal thickness in minipigs.

So far, no published studies have investigated VDR agonist-induced effects on HA in the skin. Our in vitro results indicate that calcipotriol has differential effects on HA in fibroblasts and keratinocytes. Calcipotriol increased HA secretion and counteracted the betamethasone-induced decrease in keratinocytes. In contrast, *HAS2* expression and HA secretion in fibroblasts remained unaffected by calcipotriol. TGF-beta has also been reported to induce *HAS2* expression in human dermal fibroblasts [6, 22]. The reported glucocorticoid-mediated decrease in TGF-beta expression correlates with the betamethasone-induced downregulation of *HAS2* in fibroblasts whereas no correlation is observed between the reported effect of calcipotriol on TGF-beta expression and lack of effect on HA secretion in fibroblasts.

Similarly, in the skin explant model calcipotriol restored betamethasone-decreased HAS-2 expression in the epidermis and furthermore prevented betamethasone-induced epidermal thinning in minipigs upon treatment with the calcipotriol/betamethasone gel. Interestingly, another study have reported that intermediate-size HA fragments induced keratinocyte proliferation in vitro as well as epidermal hyperplasia in vivo and reversed glucocorticoid-induced epidermal thinning in humans [16].

A previous clinical study in healthy human volunteers has investigated the effect of the calcipotriol/betamethasone combination on skin thickness as assessed by sonography [31]. In this study, the combination treatment reduced total skin thickness by approximately 15 % after 4 weeks of treatment comparable to the effect of betamethasone dipropionate alone. The treatment effect was confined to the dermis as no treatment effect was seen on epidermal thickness. The reduction in skin thickness observed in this clinical study can maybe be explained by a rapid loss of water binding capacity in the dermis due to a decrease of HA. This is further supported by the fast recovery of skin thickness observed upon termination of treatment. The reported reduction in dermal thickness by calcipotriol/betamethasone is in contrast to our finding that calcipotriol counteracts betamethasone-induced decrease in collagen I synthesis. However, it is likely that the 4 week duration of the clinical study was too short as to reveal a counteracting effect of calcipotriol on dermal thickness via a stimulation of collagen production.

In summary, our results overall confirm the previously reported atrophogenic effect of glucocorticoids via decrease of key ECM components in the skin. Of notice, we show for the first time in primary human skin cultures that calcipotriol reduces early signs of betamethasone-induced skin atrophy by modulation of ECM components. These results provide a probable mechanism behind a favorable safety profile of the two-compound fixed-combination gel for topical treatment of psoriasis.

## Electronic supplementary material

Below is the link to the electronic supplementary material.

Supplementary material 1 (PDF 487 kb)

Supplementary material 2 (PDF 431 kb)

Supplementary material 3 (PDF 705 kb)

Supplementary material 4 (PDF 1916 kb)

Supplementary material 5 (PDF 948 kb)
