# Supplementary material for: Calcipotriol counteracts betamethasone-induced decrease in extracellular matrix components related to skin atrophy
Source: Arch Dermatol Res. 2014 Jul 16;306(8):719–29. doi: 10.1007/s00403-014-1485-3 (PMC4168021; doi:10.1007/s00403-014-1485-3)
Supplement: Supplementary file 1 — Supplementary material 1 (PDF 487 kb) [file 403_2014_1485_MOESM1_ESM.pdf]

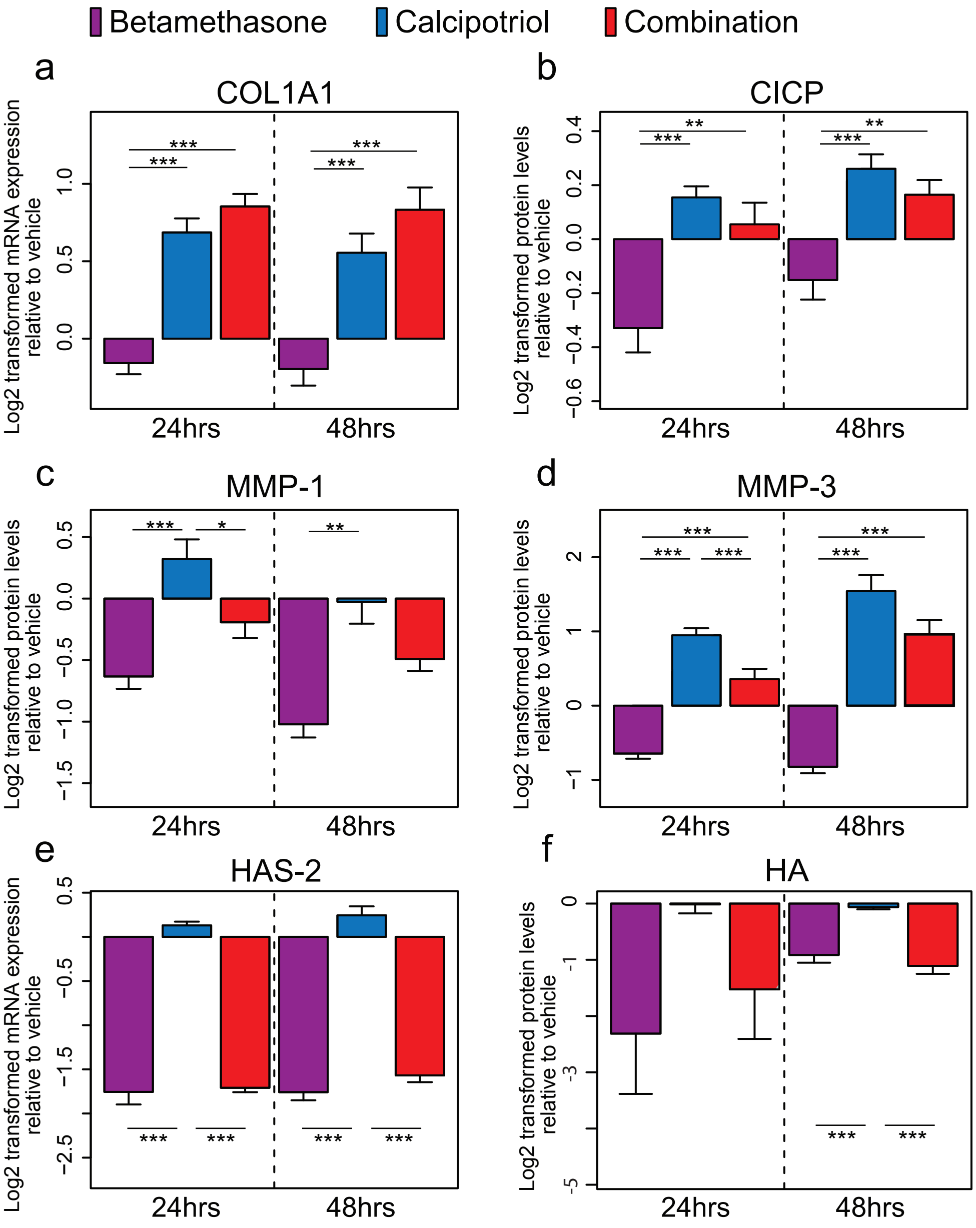

Supplementary Fig. 1 Calcipotriol counteracts betamethasone-induced suppression of pro-collagen I and MMP-1 and-3 but not HA production in primary human dermal fibroblasts. Cultures were treated in duplicates as indicated and analyzed for (a) COL1A1 mRNA expression, (b) CICP (C-terminal propeptide of type I collagen), (c) MMP-1 and (d) MMP-3 from culture supernatants, (e) HAS2 mRNA expression and (f) HA from culture supernatants. qPCR measurements for COL1A1 and HAS2 were normalized to GAPDH. Data were log-transformed and scaled to vehicle within each experiment. Values represented are means ± SEM from three (qPCR) and four (protein analyses) independent experiments using two donors. P-values were determined with a Repeated Measures One-way ANOVA Test followed by Tukey's Multiple Comparison Test (R version 2.15.2) and indicated with * P<0.05; ** P<0.01; *** P<0.001.

Arch. Dermatol. Res.

Calcipotriol counteracts betamethasone-induced decrease in extracellular matrix components related to skin atrophy.

Hanne Norsgaard[1], Sandrine Kurdykowski[5], Pascal Descargues[5], Tatiana Gonzalez[2], Troels Marstrand[1], Georg Dünstl[3], and Mads Røpke[4].

Department of [1]Molecular Biomedicine, [2]Disease Pharmacology, [3]External Discovery, and [4]Clinical Pharmacology, LEO Pharma A/S, Industriparken 55, Ballerup, Denmark. [5]Genoskin, Oncopole, 1 place Pierre Potier, Toulouse, France.

e-mail: hanne.norsgaard@leo-pharma.com
