# Supplementary material for: Calcipotriol counteracts betamethasone-induced decrease in extracellular matrix components related to skin atrophy
Source: Arch Dermatol Res. 2014 Jul 16;306(8):719–29. doi: 10.1007/s00403-014-1485-3 (PMC4168021; doi:10.1007/s00403-014-1485-3)
Supplement: Supplementary file 2 — Supplementary material 2 (PDF 431 kb) [file 403_2014_1485_MOESM2_ESM.pdf]

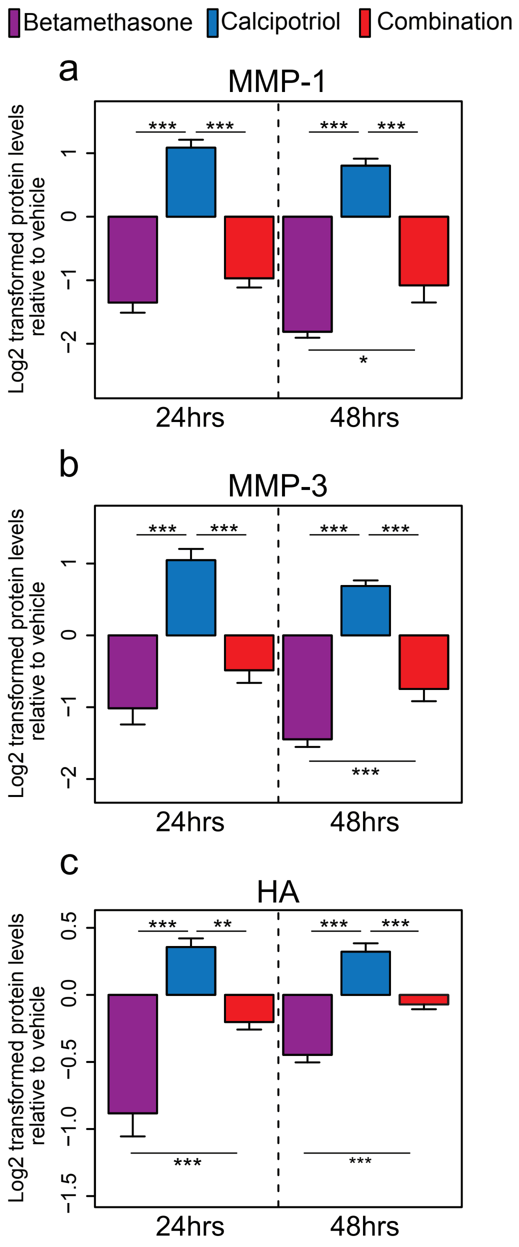

Supplementary Fig. 2 Calcipotriol counteracts betamethasone-induced reduction of MMP-1, MMP-3 and HA production in primary human epidermal keratinocytes. Differentiation was induced by 1.2 mM calcium and cultures were treated in duplicates as indicated and analyzed for (a) MMP-1, (b) MMP-3 and (c) HA from culture supernatants. Data were log-transformed and scaled to vehicle within each experiment. Values represented are means ± SEM from five independent experiments using three donors. P-values were determined with a Repeated Measures One-way ANOVA Test followed by Tukey's Multiple Comparison Test (R version 2.15.2) and indicated with * P<0.05; ** P<0.01; *** P<0.001.

Arch. Dermatol. Res.

Calcipotriol counteracts betamethasone-induced decrease in extracellular matrix components related to skin atrophy.

Hanne Norsgaard[1], Sandrine Kurdykowski[5], Pascal Descargues[5], Tatiana Gonzalez[2], Troels Marstrand[1], Georg Dünstl[3], and Mads Røpke[4].
Department of [1]Molecular Biomedicine, [2]Disease Pharmacology, [3]External Discovery, and [4]Clinical Pharmacology, LEO Pharma A/S, Industriparken 55, Ballerup, Denmark. [5]Genoskin, Oncopole, 1 place Pierre Potier, Toulouse, France.

e-mail: hanne.norsgaard@leo-pharma.com
