# Supplementary material for: Calcipotriol counteracts betamethasone-induced decrease in extracellular matrix components related to skin atrophy
Source: Arch Dermatol Res. 2014 Jul 16;306(8):719–29. doi: 10.1007/s00403-014-1485-3 (PMC4168021; doi:10.1007/s00403-014-1485-3)
Supplement: Supplementary file 3 — Supplementary material 3 (PDF 705 kb) [file 403_2014_1485_MOESM3_ESM.pdf]

a

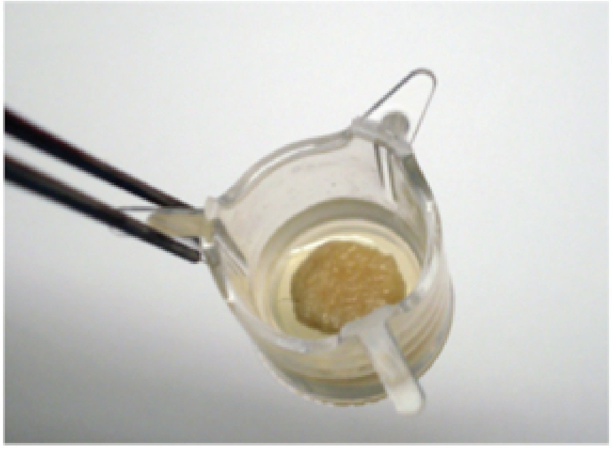

b

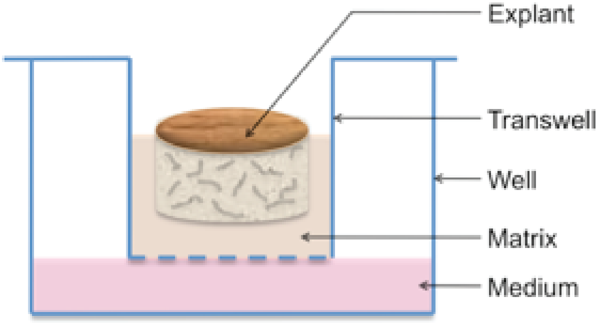

Supplementary Fig. 3 NativeSkin® model. (a) Macroscopic visualization and (b) schematic representation of the human skin explant model.

Arch. Dermatol. Res.

Calcipotriol counteracts betamethasone-induced decrease in extracellular matrix components related to skin atrophy.

Hanne Norsgaard[1], Sandrine Kurdykowski[5], Pascal Descargues[5], Tatiana Gonzalez[2], Troels Marstrand[1], Georg Dünstl[3], and Mads Røpke[4].
Department of [1]Molecular Biomedicine, [2]Disease Pharmacology, [3]External Discovery, and [4]Clinical Pharmacology, LEO Pharma A/S, Industriparken 55, Ballerup, Denmark. [5]Genoskin, Oncopole, 1 place Pierre Potier, Toulouse, France.

e-mail: hanne.norsgaard@leo-pharma.com
