# Supplementary material for: Calcipotriol counteracts betamethasone-induced decrease in extracellular matrix components related to skin atrophy
Source: Arch Dermatol Res. 2014 Jul 16;306(8):719–29. doi: 10.1007/s00403-014-1485-3 (PMC4168021; doi:10.1007/s00403-014-1485-3)
Supplement: Supplementary file 4 — Supplementary material 4 (PDF 1916 kb) [file 403_2014_1485_MOESM4_ESM.pdf]

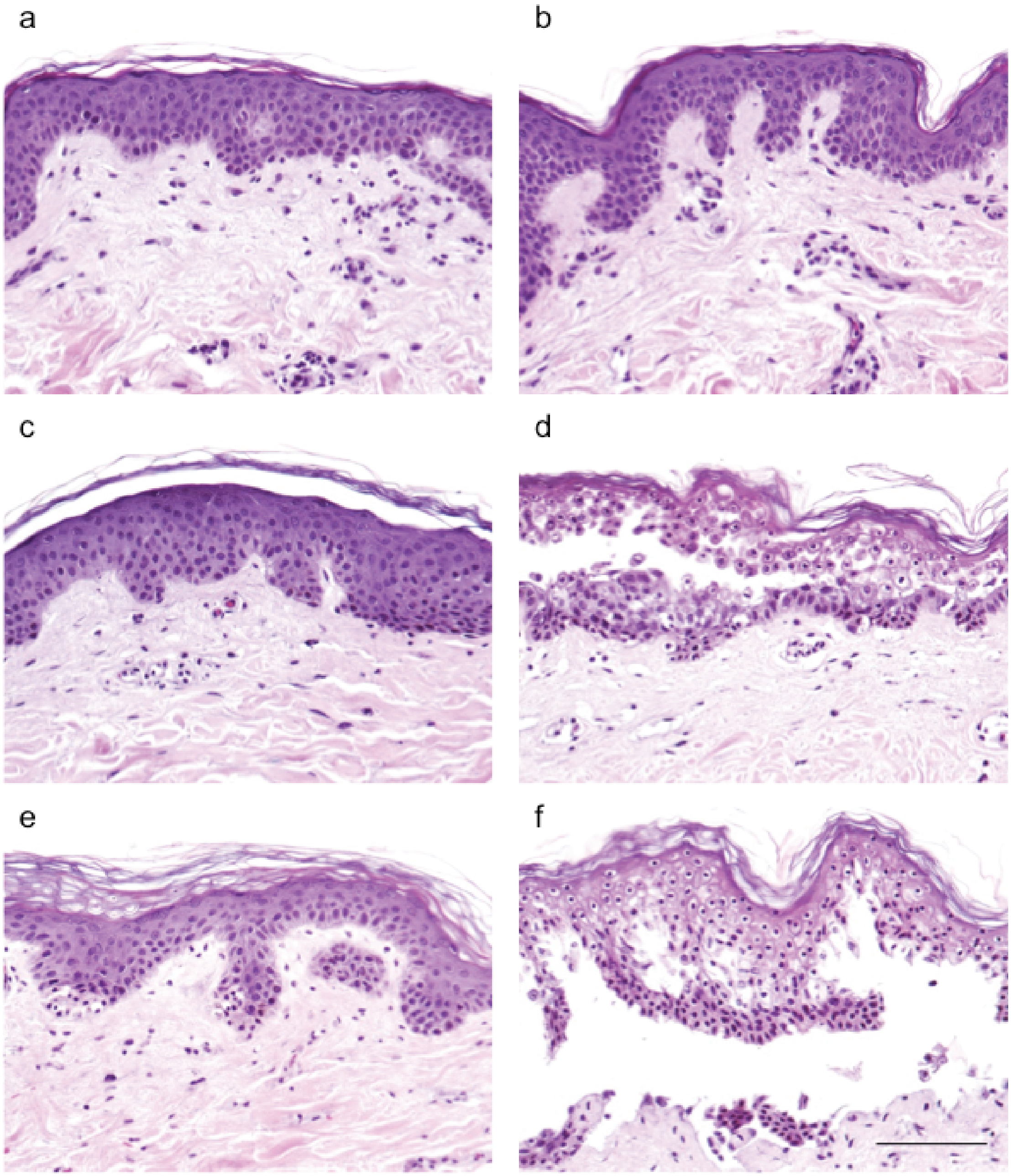

Supplementary Fig. 4 The matrix of the NativeSkin® model maintains tissue integrity and viability. Hematoxylin and eosin staining of skin cross sections at day 1 (a and b), day 3 (c and d) and day 7 (e and f) of *ex vivo* culture with proprietary culture medium using skin from the same donor. Panels a, c and e correspond to NativeSkin® technology (skin biopsies inserted into a proprietary matrix) whereas panels b, d and f correspond to skin biopsies left on the porous membrane of a cell culture insert during the *ex vivo* culture. Scale bar is 100 µm for all panels.

Arch. Dermatol. Res.

Calcipotriol counteracts betamethasone-induced decrease in extracellular matrix components related to skin atrophy.

Hanne Norsgaard[1], Sandrine Kurdykowski[5], Pascal Descargues[5], Tatiana Gonzalez[2], Troels Marstrand[1], Georg Dünstl[3], and Mads Røpke[4].

Department of [1]Molecular Biomedicine, [2]Disease Pharmacology, [3]External Discovery, and [4]Clinical Pharmacology, LEO Pharma A/S, Industriparken 55, Ballerup, Denmark. [5]Genoskin, Oncopole, 1 place Pierre Potier, Toulouse, France.

e-mail: hanne.norsgaard@leo-pharma.com
