# Supplementary material for: Calcipotriol counteracts betamethasone-induced decrease in extracellular matrix components related to skin atrophy
Source: Arch Dermatol Res. 2014 Jul 16;306(8):719–29. doi: 10.1007/s00403-014-1485-3 (PMC4168021; doi:10.1007/s00403-014-1485-3)
Supplement: Supplementary file 5 — Supplementary material 5 (PDF 948 kb) [file 403_2014_1485_MOESM5_ESM.pdf]

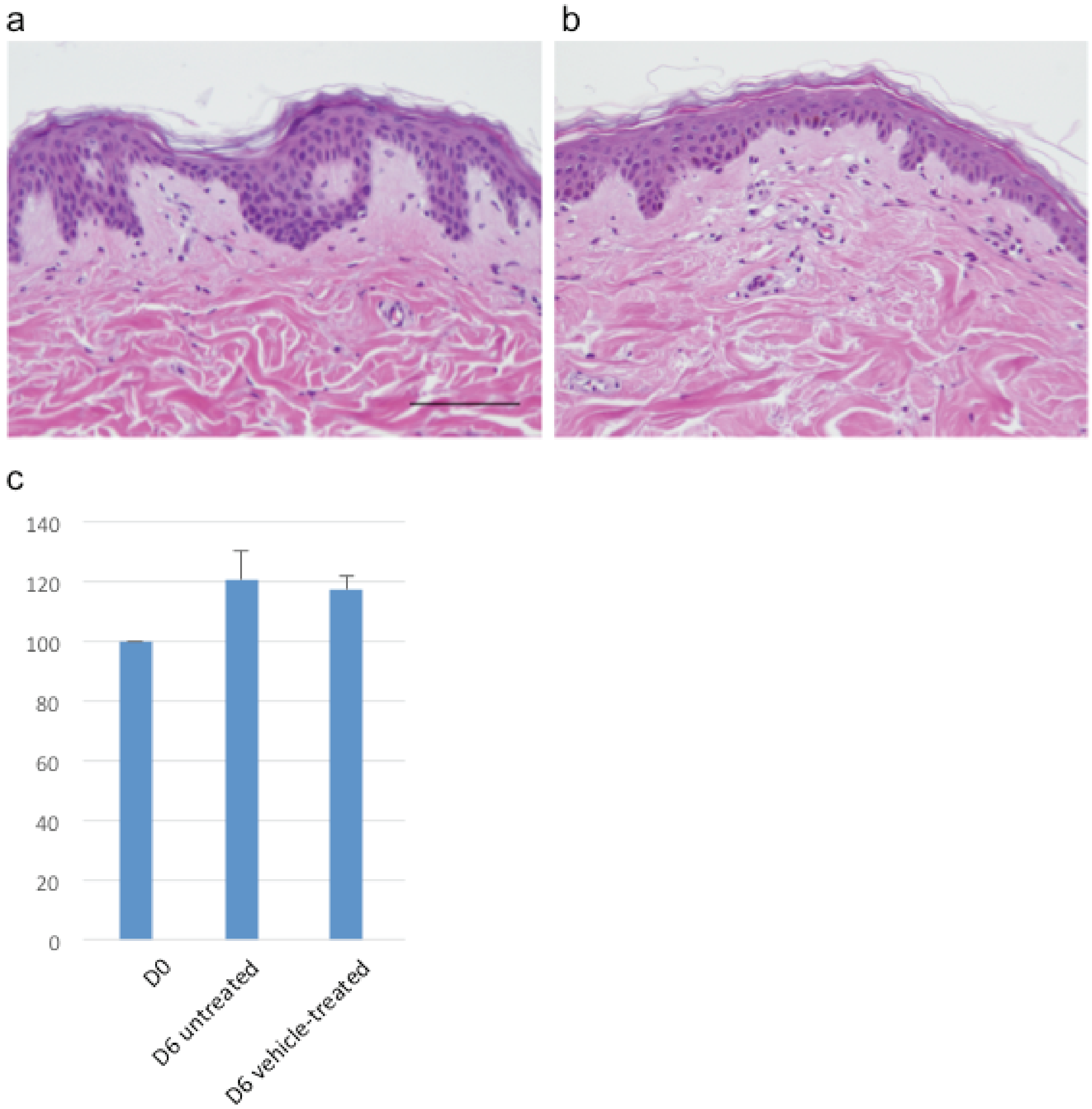

Supplementary Fig. 5 Skin morphology and epidermal layers are not affected upon repeated topical application of the vehicle gel. Hematoxylin and eosin staining of the NativeSkin® model (a) at start of treatment and (b) 6 days after *ex vivo* culture following daily topical application of the vehicle gel. Scale bars are 100 µm. (c) Mean epidermal thickness of untreated and vehicle-treated controls at day 6 in percentage of day 0. Stratum corneum was excluded in the measurements using ImageJ software.

Arch. Dermatol. Res.
Calcipotriol counteracts betamethasone-induced decrease in extracellular matrix components related to skin atrophy.
Hanne Norsgaard[1], Sandrine Kurdykowski[5], Pascal Descargues[5], Tatiana Gonzalez[2], Troels Marstrand[1], Georg Dünstl[3], and Mads Røpke[4].
Department of [1]Molecular Biomedicine, [2]Disease Pharmacology, [3]External Discovery, and [4]Clinical Pharmacology, LEO Pharma A/S, Industriparken 55, Ballerup, Denmark. [5]Genoskin, Oncopole, 1 place Pierre Potier, Toulouse, France.

e-mail: hanne.norsgaard@leo-pharma.com
